# Genetic Analysis of *mcr-1-*Carrying Plasmids From Gram-Negative Bacteria in a Dutch Tertiary Care Hospital: Evidence for Intrapatient and Interspecies Transmission Events

**DOI:** 10.3389/fmicb.2021.727435

**Published:** 2021-09-06

**Authors:** Nikolaos Strepis, Anne F. Voor in ’t holt, Margreet C. Vos, Willemien H. A. Zandijk, Astrid P. Heikema, John P. Hays, Juliëtte A. Severin, Corné H. W. Klaassen

**Affiliations:** Department of Medical Microbiology and Infectious Diseases, Erasmus MC University Medical Center, Rotterdam, Netherlands

**Keywords:** genomics, bacterial drug resistance, colistin, plasmids, transmission

## Abstract

The role of plasmids in the complex pandemic of antimicrobial resistance is increasingly being recognized. In this respect, multiple mobile colistin resistance (*mcr*) gene-carrying plasmids have been described. However, the characteristics and epidemiology of these plasmids within local healthcare settings are largely unknown. We retrospectively characterized the genetic composition and epidemiology of plasmids from *mcr-1-*positive bacterial isolates identified from patients from a large academic hospital in the Netherlands. Clinical Gram-negative bacteria with an MIC > 2 μg/mL for colistin, obtained from patients hospitalized at the Erasmus MC University Medical Center Rotterdam during the years 2010–2018, were screened for presence of the *mcr-1* gene. Extracted plasmids from *mcr-1*-positive isolates were sequenced using a combination of short- and long-read sequencing platforms, characterized by incompatibility type and genetic composition and compared to publicly available *mcr-1-*carrying plasmid sequences. In 21 isolates from 14 patients, *mcr-1* was located on a plasmid. These plasmids were of diverse genetic background involving Inc types IncX4, IncI2(delta), IncHI2, as well as double Inc types IncHI2/IncN and IncHI2/IncQ. *mcr-1*-carrying plasmids were found in *Escherichia coli, Klebsiella pneumoniae, and Kluyvera georgiana*, and within the chromosome of an ST147 *K. pneumoniae* isolate. In depth analysis indicated intrapatient, interpatient, and interspecies transmission events of *mcr-1-*carrying plasmids. In addition, our results show that the *mcr-1* gene resides in a rich environment full of other (*mcr-1* negative) plasmids and of many different Inc types, enabling interplasmidal transfer events and facilitating widespread dissemination of the *mcr-1* gene. Multiple *mcr-1*-carrying plasmid transmission events had likely occurred among isolates from hospitalized patients. Recognition and identification of plasmid transmission events within hospitals is necessary in order to design and implement effective infection control measures.

## Introduction

The plasmid-mediated mobile colistin resistance gene (*mcr-1*) was first reported in 2015 and has been detected across all continents (Liu et al., 2016; Wang R. et al., 2018; Elbediwi et al., 2019). Since then, at least 10 different *mcr* genes have been identified, with *mcr-1* being the most prevalent colistin resistance gene in humans and animals (Irrgang et al., 2016; Ye et al., 2016). The global spread of *mcr-1* is a potential challenge to the effective use of antibiotics to treat multi-drug resistant microorganisms, particularly as colistin is considered as an “antibiotic of last resort” (Gregoire et al., 2017). Additionally, the location of *mcr-1* within plasmids contributes to the gene’s mobility via horizontal transfer, thus allowing the spread of colistin resistance between different bacterial species. This phenomenon has been observed in livestock and more recently also in healthcare settings (Carattoli, 2013; Liu et al., 2016; Shen et al., 2018; Wang X. et al., 2018).

To date, *mcr* genes have been detected in plasmids of diverse incompatibility (Inc) types, with IncI2, IncHI2, and InX4 being the most abundant types identified (Matamoros et al., 2017). Plasmids carrying the *mcr-1* gene have been mainly described in *Escherichia coli* and *Klebsiella pneumoniae* (Terveer et al., 2017; Wang et al., 2017), but have also been identified in other Enterobacterales (Tse and Yuen, 2016; Caselli et al., 2018). From a large study in Parisian hospitals, based on the genetic background of the strains, it was suggested human isolates carrying an *mcr-1* plasmid may have an animal origin (Bourrel et al., 2019). In addition, nosocomial spread of the *mcr-1* gene or *mcr-1*-containing plasmids has been rarely reported (Mendes et al., 2018; Mariani et al., 2020). This may be due to the current limited use of plasmid detection and characterization for hospital epidemiology and infection prevention and control purposes.

In this study, we analyzed the epidemiology and genetics of *mcr-1*-carrying plasmids isolated from patients admitted to a large tertiary care hospital in the Netherlands. We characterized plasmids responsible for the spread of *mcr-1* in colistin-resistant bacteria isolated from patients and compared these to publicly available plasmid sequences. As a result, we were able to observe inter- and intrapatient transmission events as well as interspecies transmission events.

## Materials and Methods

### Study Design

This retrospective observational study was conducted at the Erasmus MC University Medical Center (Erasmus MC) in Rotterdam, the Netherlands. Highly resistant microorganisms (HRMO) and non-HRMO isolates (stored at −80°C) known not to be intrinsically resistant to colistin, from different collections, were included in the study (Supplementary File 1). Bacterial isolates collected from January 2010 until September 2018 were included in the study (Supplementary File 1). Enterobacterales, as well as *Acinetobacter baumannii-calcoaceticus* complex and *Pseudomonas aeruginosa* isolates were included (Supplementary File 1), all with an MIC > 2 μg/mL for colistin (as determined by the Vitek 2^®^ system – bioMérieux, Marcy-l’Étoile, France). The applied cut-off value was validated by also screening isolates with an MIC ≤ 2 (Supplementary File 1). Approval to conduct the study was received from the medical ethics research committee of the Erasmus MC (MEC-2015-306).

### Total DNA Extraction

A 1 μL loop of bacterial inoculum was suspended in 200 μL phosphate-buffered saline (PBS) solution. DNA extraction was performed using a MagNA Pure 96 (Roche Diagnostics, Basel, Switzerland) and the MagNA Pure DNA/Viral NA SV 2.0 kit with the Pathogen Universal 200 protocol. Total genomic DNA was subjected to amplified fragment length polymorphism (AFLP) fingerprinting (van Burgh et al., 2019) using two optimized conditions for *E. coli* with restriction enzymes *Hpy*CH4IV and *Mse*I using as selective residues C + GT and G + GA, respectively.

### Real-Time PCR

The presence of the *mcr-1* gene was determined using real-time PCR. Five μL of purified DNA, amplification primers 5′-GGTGGGTAAGCTTGCCAGTA-3′ (0.5 μM), 5′-GCGTCTTTGGCGTGATAAAT-3′ (0.5 μM) and probe 5′-FAM-AAAAGCCAGTGCGCCAAAAGATACC-BHQ-3′ (0.2 μM) (Eurogentec, Liège, Belgium) were combined in 20 μL amplification reactions in 1x LightCycler^®^ 480 Probes Master (Roche Diagnostics). Thermocycling consisted of an initial denaturation for 5 min at 95°C followed by 50 cycles of 5 s at 95°C and 30 s at 60°C, performed on a LightCycler^®^ 480 instrument (Roche Diagnostics). *mcr-1*-positive and negative control strains were included in each run.

### Plasmid Extraction

A 10 μL loop of bacterial inoculum was suspended in 200 μL PBS solution. Plasmid DNA extraction was performed using the Bioline plasmid miniprep kit (Bioline, Ranst, Belgium). The protocol of the manufacturer was followed for low copy number plasmids including all optional steps, although the speed of all centrifugation steps was reduced to 4,000 × *g* (except the initial centrifugation step for the lysis of the bacterial cells, which was maintained at 16,000 × *g*). DNA concentration was measured using the Quant-iT PicoGreen dsDNA Assay Kit (Thermo Fisher Scientific, Waltham, MA, United States).

### Total and Plasmid DNA Sequencing

Extracted plasmid DNA and/or total DNA were sequenced using Illumina and Nanopore sequencing platforms. Illumina sequencing library preparation was conducted with the NextEra DNA Flex Library Prep Kit (Illumina, San Diego, CA, United States). Sequencing was conducted using the Illumina iSeq 100 System (Illumina) generating 150 bp paired end reads. Library preparation for Nanopore sequencing was performed using the Rapid Barcoding Sequencing Kit SQK-RBK004 (Oxford Nanopore Technologies, Oxford, United Kingdom) according to the standard protocol described by the manufacturer.

### Sequencing Data Assembly and Genome Annotation

Fastq files from Illumina and Nanopore sequencing were combined in a hybrid assembly using Unicycler v0.4 (Wick et al., 2017) with default parameters and visualized using Bandage (Wick et al., 2015). The assembled plasmid sequences were further validated by read mapping and visual checking for assembly errors. Plasmid sequences were annotated using Prokka v1.13 (Seemann, 2014). Syntenic regions were visualized using Geneious v2019.1 (Biomatters, Auckland, New Zealand). Whole plasmid alignment was performed using the progressiveMauve algorithm from Mauve 3.6 (Darling et al., 2004). Plasmidfinder (Carattoli et al., 2014) was used to identify plasmid Inc type(s). In order to distinguish between large plasmids and a possible chromosomal location of the *mcr-1* gene, selected bacterial isolates were additionally sequenced via Nanopore sequencing on total genomic DNA. The presence of antibiotic resistance genes/mechanisms were identified using a stand-alone version of RGI (Jia et al., 2017) v5.1.0 based on the CARD database v3.0.5 (including Perfect and Strict hits) and ResFinder (Zankari et al., 2012) 3.2 (database 2019-10-01). Mobilization module characterization was based on MOBscan (Garcillán-Barcia et al., 2020) and the insertion elements were identified based on ISfinder (Siguier et al., 2006).

The NCBI genomic repository was searched for *mcr-1* containing sequences^1^ (2019-05-07) ignoring sequences shorter than 1.5 kbp or larger than 1 Mbp as well as ignoring sequences that were annotated as (cloning) vectors and sequences lacking an Inc type. Search results were further filtered by the presence of *mcr-1* by a BLAST search (49) against the *mcr-1* sequence (MH143576.1) using default parameters. kSNP v3.01 (Gardner et al., 2015) was used for k-mer based SNP analysis of plasmid sequences of similar Inc types. K-mer chooser was used to define the optimal k-mer value for each Inc type. ITOL (Letunic and Bork, 2016) was used for visualizing the maximum likelihood trees based on core and accessory SNPs. Integron identification was performed using Integron Finder v2.0 (Cury et al., 2016). When applicable, the multi locus sequence type (MLST) was identified based on each species’ corresponding MLST scheme (Jolley et al., 2018).

## Results

### Patient and Strain Characteristics

Three hundred and twenty-nine bacterial isolates were screened from a total of 270 patients. Ninety-six isolates (29.2%) were described as extended-spectrum beta-lactamase (ESBL)-positive, and eight isolates (2.4%) were stored because of the presence of a carbapenemase gene (Supplementary File 1). Real-time PCR demonstrated the presence of the *mcr-1* gene in 22 isolates, derived from 14 individual patients The oldest *mcr-1* positive isolates in this collection dated back to two patients admitted in 2010. Fourteen out of the 22 *mcr-1* positive isolates were ESBL-positive and all isolates were imipenem and meropenem susceptible. The *mcr-1* positive samples included isolates of *E. coli* (*n* = 19), *K. pneumoniae* (*n* = 2) and *Kluyvera georgiana* (*n* = 1), and were cultured from rectum (*n* = 13), urine (*n* = 6), throat (*n* = 1), vagina (*n* = 1), and blood (*n* = 1).

Eight out of 14 patients were male (57.1%), and the mean age was 51 (range 12–73). Five adult patients and two pediatric patients were treated for a hematologic disease at the moment of their positive culture. None of the 14 patients died within 28 days after the first positive culture. Out of the 14 patients, seven patients were previously admitted to the Erasmus MC, and two patients were hospitalized abroad within 1 year before the positive culture. No additional *mcr-1* positive isolates were identified when screening isolates with an MIC ≤ 2 (Supplementary File 1).

### Plasmid Architecture

Hybrid assembly of short and long sequencing reads generated multiple plasmid sequences per isolate (Tables 1, 2 and Supplementary Table 1).

**TABLE 1:** Plasmids carrying *mcr-1* present in the patient isolates.

**Plasmid no.**	**Patient**	**Year**	**Species**	**Inc type(s)**	**MOB modules**	**Size (bp)**
Plasmid 1	A	2010	*E. coli*	IncX4•	MOBP	30,828
Plasmid 2	B	2010	*E. coli*	IncHI2/IncQ1•	MOBH	262,507*
Plasmid 3	C	2014	*E. coli^2^*	IncX4•	MOBP	33,309
Plasmid 4	C	2014	*E. coli^2^*	IncX4•	MOBP	33,309
Plasmid 5	D	2015	*E. coli^1^*	IncI2(delta)	MOBP	57,198
Plasmid 6	E	2015	*K. pneumoniae*	IncI2(delta)•	MOBP	62,627
Plasmid 7	E	2015	*K. georgiana*	IncI2(delta)•	MOBP	64,053
Plasmid 8	E	2015	*E. coli*	IncI2(delta)•	MOBP	64,066
Plasmid 9	F	2015	*E. coli*	IncHI2		54,022
Plasmid 10	G	2015	*E. coli^3^*	IncHI2/IncQ1•	MOBH	296,174*
Plasmid 11	H	2016	*E. coli*	IncI2(delta)	MOBP	60,958
Plasmid 12	H	2016	*E. coli*	IncI2(delta)	MOBP	57,430
Plasmid 13	D	2016	*E. coli^1^*	IncI2(delta)	MOBP	64,047
Plasmid 14	D	2016	*E. coli*	IncI2(delta)•	MOBP	64,632
Plasmid 15	E	2016	*E. coli*	IncI2(delta)	MOBP	62,805
Plasmid 16	I	2016	*E. coli*	IncX4•	MOBP	33,303
Plasmid 17	G	2016	*E. coli^3^*	IncHI2		54,236
Plasmid 18	J	2017	*E. coli*	IncX4	MOBP	33,310
Plasmid 19	K	2017	*E. coli*	IncHI2/IncQ1•	MOBH	206,896*
Plasmid 20	L	2017	*E. coli*	IncHI2/IncN•	MOBH	257,516*
Plasmid 21	M	2017	*E. coli*	IncX4•	MOBP	33,066

Genotypically indistinguishable isolates are defined by superscript numbers attached to the species name. • Complete circular plasmid. *Plasmids assembled from total genomic DNA.

**TABLE 2:** Inc types of *mcr-1* plasmids identified in public datasets and from this study.

**Inc type**	**Public**	**This study**
IncFII	1(0.32%)	0(0.00%)
IncHI2	54(17.00%)	3(13.63%)
IncI2	87(28.00%)	0(0.00%)
IncI2(delta)	44(14.37%)	10(45.45%)
IncR	1(0.32%)	0(0.00%)
IncX4	84(27.45%)	6(27.27%)
IncY	5(1.63%)	0(0.00%)
Multi-Inc	27(8.82%)	3(13.63%)
p0111	3(0.08%)	0(0.00%)

**FIGURE 1:**
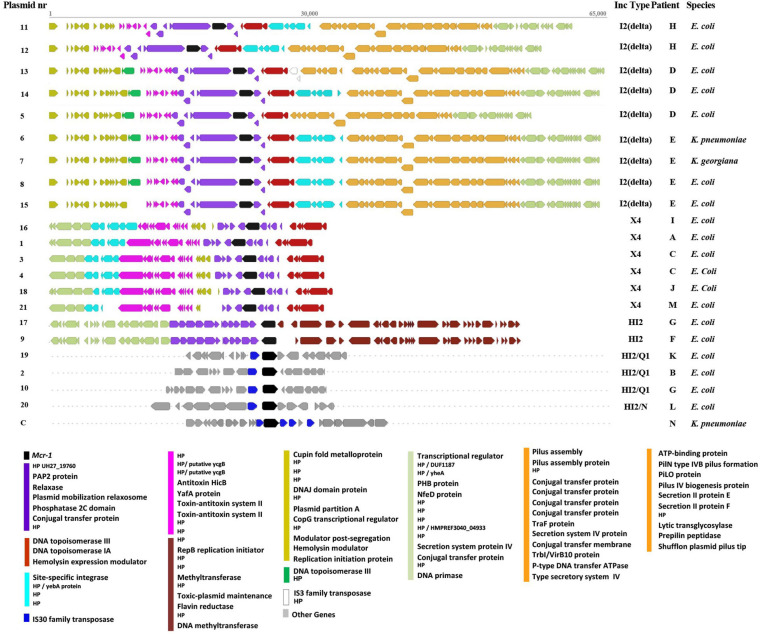
Graphical representation of genomic regions in plasmids containing the *mcr-1* gene and annotated using Prokka. Conserved regions as defined by Mauve alignment are represented by different colors. Each arrow represents a gene and dots indicate that the presented region is part of a much larger plasmid/chromosome. *HP*, hypothetical protein encoding gene.

All *mcr-1* genes encoded the *mcr*-1.1 variant. The *mcr-1* gene was surrounded by different genes in different Inc type plasmids, but for each Inc type there were remarkable similarities. MOBscan analysis indicated the presence of relaxases encoding genes of MOBH (Plasmid 2, 10, 19, and 20) and MOBP (Plasmid 1, 3, 4, 5, 6, 7, 8, 11, 12, 13, 14, 15, 16, 18, and 21). For two plasmids (9 and 17) no result was obtained. Analysis of *mcr-1* plasmid architectures revealed regions with similarities among the plasmids (Figure 1), with a conserved group of 23 genes – dedicated to pili formation and conjugal transfer – in IncI2(delta) *mcr-1* plasmids (Figure 1).

Although two IncHI2 plasmids were detected of approximately 55 kbp and 206 kbp, respectively, three other *mcr-1* positive IncHI2 plasmids were of a double Inc type. More specifically, in two cases a plasmid with Inc type IncHI2 had merged with another plasmid carrying either IncQ or IncN, resulting in a large plasmid (>200 kbp) of double Inc type (Table 1). These double Inc type *mcr-1-*carrying plasmids clustered independently from each other and from the plasmids with single IncHI2 type in SNP analysis. While IncI2 is observed as one of the most commonly identified *mcr-1*-carrying plasmids in public repositories, in our samples we only detected IncI2(delta), as the most prevalent Inc type in our study and no IncI2 was found (Table 2). Three novel additional multi-Inc types plasmids were identified in this study, adding to the known (multi-)Inc type *mcr-1*-carrying plasmids.

In *mcr-1*-carrying plasmids, additional antibiotic resistance genes were identified. The *bla*_CTX–M_ gene encoding CTX-M-64 was detected in five IncI2(delta) plasmids and encoding CTX-M-14 was detected in two IncHI2 *mcr-1* plasmids (Supplementary Table 2). Plasmid 20 carried seven antibiotic resistance genes including a *bla*_TEM–1_ and *aadA* while plasmid 2 carried a large number of 21 antibiotic resistance genes, including multiple aminoglycoside antibiotic resistance genes. In addition, plasmids 20 and 2 carried heavy metal resistance genes, with plasmid 20 possessing genes for copper resistance and two operons; one of cation efflux system proteins, and one of four copper resistance proteins. Plasmid 2 carried an operon related to mercury resistance involving mercuric transport proteins and a mercury reductase protein).

In addition to the *mcr-1*-carrying plasmids, up to eight (average four) other plasmids were identified per strain. The size range of these additional plasmids was 1.3–163 kbp (Supplementary Table 1). Twenty different Inc types were identified in the non-*mcr-1* plasmids including the Inc types that have already been described in *mcr-1* positive plasmids. Another double Inc type plasmid was observed (IncFIA/IncFIB) in an *E. coli* isolate.

### Plasmid Transmissions in Patients

**FIGURE 2:**
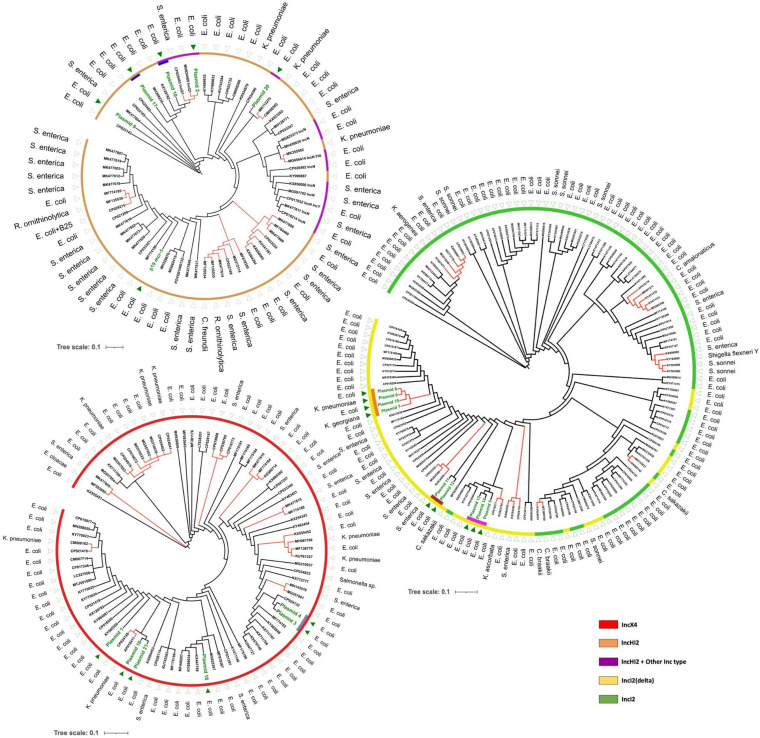
Maximum likelihood trees based on SNP analysis (core and accessory SNPs) of *mcr-1*-carrying plasmids using k-mers defined by kSNP. Separate figures are provided for plasmids of IncX4, IncI2/IncI2(delta), and IncHI2 (including IncHI2/IncQ1 and IncHI2/IncN). Dark green text and triangles are plasmids from this study isolates; colored bars indicate plasmids from the same patient, the color of the outer circles correspond to Inc types. Red branches indicate plasmid clusters involving different bacterial species.

A comparison of plasmids in this study with publicly available plasmid sequences was made to investigate whether there was transmission or multiple independent acquisitions of similar/identical plasmids between and within patients. A total of 314 plasmid sequences from GenBank were available for comparison (Table 1). Plasmids of selected Inc types were compared in a SNP analysis by kSNP (Figure 2). In general, public plasmid sequences were very different from plasmids from our study as the latter ones formed completely separate branches in the dendrograms.

**FIGURE 3:**
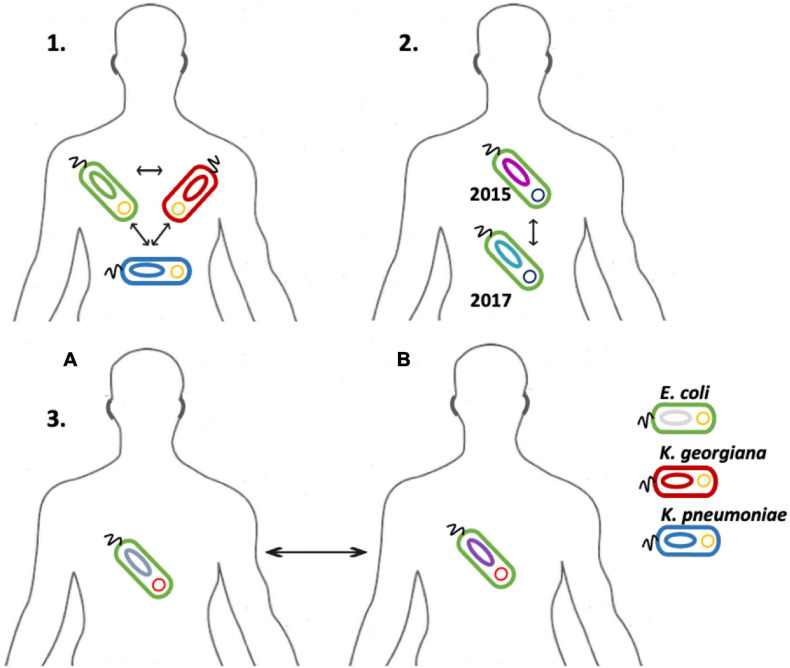
Illustration of the transmission events detected in this study’s dataset. The possible plasmid transfer among species is depicted in three separate cases. Case 1, an IncI2(delta) *mcr-*1-carrying-plasmid has been transferred among three different species of *E. coli, K. pneumoniae* and *K. georgiana* in a patient. Case 2, an IncI2(delta) *mcr-*1-carrying-plasmid was transferred among two different *E. coli* strains, one isolate detected at 2015 and one at 2017 in the same patient. Case 3, an IncX4 *mcr-*1-carrying-plasmid was transferred among two different patients in two different *E. coli* strains.

Although our dataset was relatively small, we observed multiple occasions of plasmid transfer within and between bacterial species. First, the most striking observation involved plasmids 6, 7, and 8 that were identical *mcr-1* carrying plasmids of IncI2(delta), and which were isolated from three different species including an *E. coli*, a *K. pneumoniae*, and a *K. georgiana* isolate cultured from the same patient over a 3-day period in 2015 – a clear indication of interspecies plasmid transmission (Table 1 and Figure 3). *K. georgiana* and *K. pneumoniae*, while a colistin MIC of >8, were isolated from a rectum sample, and *E. coli*, while a colistin MIC of 8, was isolated from a throat sample. All three isolates produced ESBL. Additionally, the same patient carried another genotypically different *E. coli* also containing an IncI2(delta) *mcr-1*-carrying plasmid (plasmid nr 15). This plasmid was closely related to the other plasmids isolated from this patient but lacked the DNA topoisomerase III gene that was found in the other plasmids (Figure 1). All four plasmids from this patient grouped together in a single branch well separated from other plasmids indicating the plasmids to be identical by descent (plasmids 6, 7, and 8 share 100% sequence identity while with plasmid 15 is 99.8% identical).

Second, we identified three matching IncI2(delta) plasmids (plasmids 5, 13, and 14) hosted by three *E. coli* isolates from the same patient (Figure 3). Plasmid 5 was from an isolate of 2015, plasmid 13 was from a genotypically indistinguishable isolate from 2016 and plasmid 14 from a genotypically unrelated isolate from 2016. All three plasmids clustered closely together in SNP analysis, although the two most recent ones carried a genomic region containing a site-specific integrase gene and hypothetical proteins that were absent in the 2015 isolate (Figure 1). In addition, plasmid 14 was lacking the IS*3* transposase gene that was found in plasmid 13.

Interestingly, we detected double Inc type *mcr-1* plasmids in *E. coli* isolates cultured from patients hospitalized in the years 2010, 2015, and 2017. An IncHI2/IncQ1 plasmid (plasmid 10) was identified in one of two identical *E. coli* isolates colonizing the same patient in two different time periods (2015 and 2016). The second *E. coli* isolate from this patient contained a different IncHI2 plasmid (plasmid 17), with a gene content similar to an IncHI2 plasmid (plasmid 9) originating from a different *E. coli* isolate cultured from a different patient in 2015 (Table 1). In contrast, all three IncHI2 plasmids clustered separate from each other and from other plasmids. Another IncHI2/IncQ1 plasmid (plasmid 2) was detected from an *E. coli* isolate cultured from a patient hospitalized in 2010.

In one isolate, the *mcr*-1 gene was not carried on a plasmid but was found in the chromosome. In the *K. pneumoniae* isolate of ST147, the *mcr-1* gene was surrounded by a hypothetical protein encoding gene and three IS*Apl1* insertion elements (IS*30* family transposases) (Li et al., 2017; Poirel et al., 2017) in a 6.7 kbp genomic region (Figure 1). A Blast search indicated that only the sequence surrounding this 6.7 kbp region matched the chromosome of several other *K. pneumoniae* isolates (Supplementary Figure 1).

All plasmid sequences were also screened for insertion sequences using ISfinder software (Supplementary Table 4). In all IncX4 plasmids a single insertion sequence was found (IS26). In four plasmids of the IncI2(delta) type no known insertion sequences were found and in four IncI2(delta) plasmids only an ISEnca1 was found. In the remaining plasmid of the IncI2(delta) type two IS elements were found. In contrast, in the larger plasmids of IncHI2 type up to 20 insertion sequences were identified with several insertion sequences occurring multiple times. This involved the previously mentioned IS*Apl1* and many others. Only few insertion sequences flanked the *mcr-1* gene (arbitrarily set at ≤3 kbp distance), this only involved IncHI2 plasmids (or IncHI2 double Inc type plasmids) and only involved IS*Apl1*.

## Discussion

A systematic screening of clinical isolates over an 8-year period to determine the prevalence of *mcr-1* resulted in a low *mcr-1* prevalence, while isolates at risk of carrying *mcr-1* were selected for inclusion in this study. Despite the low prevalence, interspecies and intrapatient plasmid-based *mcr-1* transfer events were detected, including one event involving chromosomal integration of the gene. The *mcr-1*-carrying plasmids identified were mainly of Inc types IncX4, IncI2(delta), and IncHI2, although *mcr-1* plasmids with double Inc types were also observed.

We identified several transmission events of *mcr-1* plasmids, including a unique case of interspecies intrapatient plasmid transmission involving *E. coli, K. pneumoniae* and *K. georgiana*. *K. georgiana* has not been described before as carrying such plasmid. In general, *Kluyvera* spp. are considered mainly environmental bacteria and only infrequently cause infections in humans. However, they can be reservoirs of multiple antibiotic resistance genes and are considered progenitors of cefotaximases and the *fosA3* gene (Rodriguez et al., 2018). In fact, in the genus of *Kluyvera*, an *mcr-1-*carrying plasmid was so far only reported in *Kluyvera ascorbata* (KU922754) (Zhao and Zong, 2016). This plasmid was different from the plasmid present in the *K. georgiana* and was more similar to three *E. coli* plasmids (13, 14, and 5) (Figure 2).

It is well established that *mcr-1* plasmids may efficiently spread between different species using laboratory conditions (Denervaud Tendon et al., 2017). By plotting the host species onto the dendrograms depicted in Figure 2 it can be recognized that interspecies transfer of *mcr-1* plasmids had occurred on multiple occasions *in vivo* as many examples are visible where (near-)identical plasmids were found in different species. These potential transfers of the same plasmid among species were not restricted to a particular Inc type but were observed in, IncX4-, IncI2-, IncI2(delta)-, and IncHI2-type plasmids and included *E. coli*, *K. pneumoniae*, *Salmonella enterica*, *Citrobacter freundii*, *Cronobacter sakazakii*, and *Raoultella ornithinolytica.*

Previous plasmid studies applied pairwise sequence comparison or average nucleotide identity (ANI) analysis (Dona et al., 2017; Dominguez et al., 2019) which is mainly applied to large sequences such as chromosomal comparisons. In this study, we compare plasmid sequences by combining a k-mer based analysis with a functional analysis. On the one hand k-mer based analysis can be applied to any sequence size and can indicate phylogenetic variations, and on the other hand functional analysis provides information regarding the genotype and phenotype (Salgado-Camargo et al., 2020). The functional comparison can result in multiple similarities among plasmids of same Inc type but k-mer based SNP analysis can indicate remarkable differences. Utilizing either one of these methods alone may restrict the amount of information obtained and actually prevent a full characterization of plasmids.

Relatively little is known about the stability of plasmids upon transfer between different strains or even between different species. Our analysis shows a remarkable heterogeneity between plasmids of identical Inc type indicating that attributing a possible transfer of plasmids by only addressing Inc types may only be presumptive (Mo et al., 2017; Han et al., 2018). A full characterization of plasmids better allows addressing these issues and also enables a more detailed study of the lateral and longitudinal stability of plasmids. The presence of highly similar plasmids found over a 3-year period in two different strains from two different patients suggests these plasmids to be stable in this time frame despite its transfer between isolates. Whether or not this is valid for all plasmids remains to be investigated. The exact transmission route was not identified in the bacterial isolates, but may have involved other patients, the innate hospital environment, or even routes in the community (including a common source), as well as animals.

Interestingly, in this study, we report one of the oldest confirmed cases of a clinical patient, hospitalized in 2010, with an *E. coli* isolate harboring an *mcr-1*-carrying IncX4 plasmid. The oldest *mcr-1*-carrying plasmid from a human isolate with a defined Inc type was reported in 2008 in an IncI2 plasmid carried by *Shigella sonnei* (Pham Thanh et al., 2016; Nang et al., 2019). Additionally, a plasmid of combined IncHI2/IncQ1 was reported in our study, in an *E. coli* isolate from 2010, indicating that these two Inc types merged into one plasmid more than a decade ago. These double Inc type plasmids are indicative of the complexity of *mcr-1* transmission and have been recognized before in public plasmid repositories (Kudirkiene et al., 2018). The impact of double Inc types in plasmids is currently unknown. However, it would be plausible that the range of host species able to carry such plasmids may be increased. Additionally, the results of two plasmids merging could affect plasmid fitness and/or the acquisition of multiple AMR genes.

The plasmids identified in our dataset derived from strains isolated from hospitalized patients were of the IncI2(delta) type (45% of plasmids in this study) which deviates from the public data set where IncI2(delta) represents only 14% of plasmids. In contrast, the most prevalent Inc types in the public dataset involved IncI2 (28%). This difference is most likely related to the hosts carrying the isolates where IncI2 might be more prevalent in farm- and dietary animal isolates and IncI2(delta) might be more prevalent in human clinical isolates. In contrast IncX4 appears to be equally present in this study and the public collection. The IncI2(delta) *mcr-1*-carrying plasmids contained a genomic region with numerous genes dedicated to pili formation and conjugation (Hu et al., 2019). Such a conserved region in IncI2(delta) plasmids may be connected to a higher transmission capacity considering our observations of IncI2(delta) plasmid transmissions.

Several non-*mcr-1* antibiotic resistance genes were detected within the plasmids found in our study, including genes generating resistance to fosfomycin, cephalosporin, aminoglycosides, carbapenems, and tetracycline. Interestingly, one *mcr-1*-carrying plasmid (plasmid nr 2) was detected containing 21 AMR genes, associated with resistance to nine different classes of antibiotics. The presence and potential spread of this 263 kbp plasmid within our academic medical center raises major concerns.

In addition to *mcr-1* carrying plasmids, all bacterial isolates from our study also contained plasmids of various other Inc types, which provides a rich genetic environment for inter-plasmidal transmission of the *mcr-1* gene. Furthermore, almost all Inc types from publicly available *mcr-1* plasmid sequences, were detected in non-*mcr-1*-carrying plasmids obtained in only a very small number of characterized isolates from our study, indicating that inter-plasmidal recombination events may already have taken place on a large scale. Such events may also have formed the basis for the multi-Inc type plasmids that we observed. The multi-Inc types plasmids described in this study contained a different MOB type relaxase encoding genes from the single Inc type plasmids. The identified IS*Apl1* transposase gene may play an important role in this process, as it may contribute to DNA recombination events by cutting and inserting DNA regions into target bacterial chromosomes or plasmids (Wang R. et al., 2018). Previously, it was established that the insertion sequence IS*Apl1* was responsible for the mobilization of the *mcr-1* gene (Li et al., 2017). Our results confirm presence of this mechanism in IncHI2 plasmids. However, in other Inc type plasmids this insertion sequence was not detected and only few (if any) other insertion sequence were detected. Despite the lack of an IS*Apl1* in IncI2 and IncX4 plasmids, the *mcr-1* gene was flanked by the *pap2* gene as the likely remains of Tn6330 (IS*Apl1-mcr-1-pap2*-IS*Apl1*) which is identified as a primary vehicle for *mcr-1* mobilization (Poirel et al., 2017; Snesrud et al., 2018). In the large IncHI2 plasmids only one copy of IS*Apl1* was maintained. The loss of the IS*Apl1* copy/copies after mobilization is a common event and may have contributed to the stabilization of the *mcr-1* cassette which appears to have remained intact among the plasmids of the same Inc type.

The presence of *mcr-1* on the chromosome of one of the colistin-resistant *K. pneumoniae* (ST147) bacterial isolates adds evidence to reports that the *mcr-1* gene may be found on the chromosome of several bacterial species (Falgenhauer et al., 2016; Yu et al., 2016; Singh et al., 2018). In our isolate, we identified an *mcr-1* gene region surrounded by multiple IS*Apl1* insertion elements (IS30 family). The region was identified as IS*Apl1-(mcr-1)-*IS*Apl1-*IS*Apl1-orf-*IS*Apl1* and was previously identified on a large IncHI2 plasmid (Wang W. et al., 2018). The international high-risk *K. pneumoniae* ST147 clone has been associated with the carriage of *bla*_NDM_ and *bla*_OXA–48_, but, to our knowledge, no cases of *mcr-1* carriage have been reported (Bahramian et al., 2019; Volozhantsev et al., 2019). The isolate from our study did not carry a major carbapenemase gene, but multiple other resistance mechanisms were found (Supplementary Table 3).

In 2016, it was stated that within the Dutch healthcare system screening for the presence of *mcr-1* in colistin-resistant bacteria from hospitalized patients is not indicated (Kluytmans-van den Bergh et al., 2016). However in our study, we showed evidence suggesting multiple transmission events of *mcr-1*-carrying plasmids, despite the gene only being identified from a relatively small set of *mcr-1* positive strains from a single medical center. Insufficient monitoring of *mcr-1* transfer within plasmids, bacterial species and patients may lead to misleading prevalence estimates within healthcare settings. Furthermore, *mcr-1* is a highly mobile gene, present in multiple types of plasmids with different attributes and found in many species (Elbediwi et al., 2019), which together with our findings indicates that an increased detection and monitoring of this gene is actually required, especially for high-risk clinical settings, e.g., hematology ward.

In order to facilitate epidemiological surveillance of *mcr-1* and other AMR plasmids, new tools need to be developed to allow diagnostic laboratories to detect *mcr-1* and AMR plasmid transmission events in hospitals and other relevant settings. A better understanding of the epidemiology of plasmid transmission events on local, national and global level is necessary for the development and implementation of measures to control the spread of the *mcr-1* gene.

## Data Availability Statement

The datasets presented in this study can be found in online repositories. The names of the repository/repositories and accession number(s) can be found below: https://www.ebi.ac.uk/ena, PRJEB37819.

## Author Contributions

NS performed the genomics analysis and composed the manuscript. AV performed strain selection and epidemiological study and composed the manuscript. MV assisted in patient and strain selection and supervised the clinical aspect. WZ contributed in laboratory experiments. AH and JH performed nanopore sequencing and contributed in genomics analysis. JS and CK supervised the complete study, contributed in defining the epidemiology and genomics points for the study, and contributed to the supervision of the study. All authors contributed to the article and approved the submitted version.

## Conflict of Interest

The authors declare that the research was conducted in the absence of any commercial or financial relationships that could be construed as a potential conflict of interest.

## Publisher’s Note

All claims expressed in this article are solely those of the authors and do not necessarily represent those of their affiliated organizations, or those of the publisher, the editors and the reviewers. Any product that may be evaluated in this article, or claim that may be made by its manufacturer, is not guaranteed or endorsed by the publisher.

## Footnotes

1
https://www.ncbi.nlm.nih.gov/nuccore/?term=mcr-1

## Supplementary Material

Supplementary Figure 1 Organization of the chromosomal region containing *mcr-1* in ST147 *K. pneumoniae*.

Supplementary Table 1 Overview of non-mcr*-1* plasmids including size and Inc types.

Supplementary Table 2 Table showing *mcr-1* plasmids that contained additional antibiotic resistance genes and the corresponding antibiotic classes.

Supplementary Table 3 Additional antibiotic resistance genes present in ST147 *K. pneumoniae* carrying *mcr-1* in its chromosome.

Supplementary Table 4 Overview of insertion sequences detected in the plasmids of this study. Numbers indicate the count of that IS element in the plasmid sequence. Of these, boldface numbers in red indicate the number flanking the *mcr*-1 gene (distance ≤3 kbp).

Supplementary File 1 Inclusion of isolates and patients January 2010 until September 2018 information. The graphic representation indicates the sample collection process followed in this study.
