# Supplementary material for: Genetic Analysis of mcr-1-Carrying Plasmids From Gram-Negative Bacteria in a Dutch Tertiary Care Hospital: Evidence for Intrapatient and Interspecies Transmission Events
Source: Front Microbiol. 2021 Sep 6;12:727435. doi: 10.3389/fmicb.2021.727435 (PMC8450869; doi:10.3389/fmicb.2021.727435)

**Supplementary Figure 1:** **Organization of the chromosomal region containing *mcr-1* in ST147 *K. pneumoniae* comparison with public sequences.** With arrows are represented the genes, where black is *mcr-1*, blue arrows represent ISApl1 elements, grey other chromosomic genes and red other plasmid genes. Grey regions are shared among chromosomes where red regions are only found in plasmids.


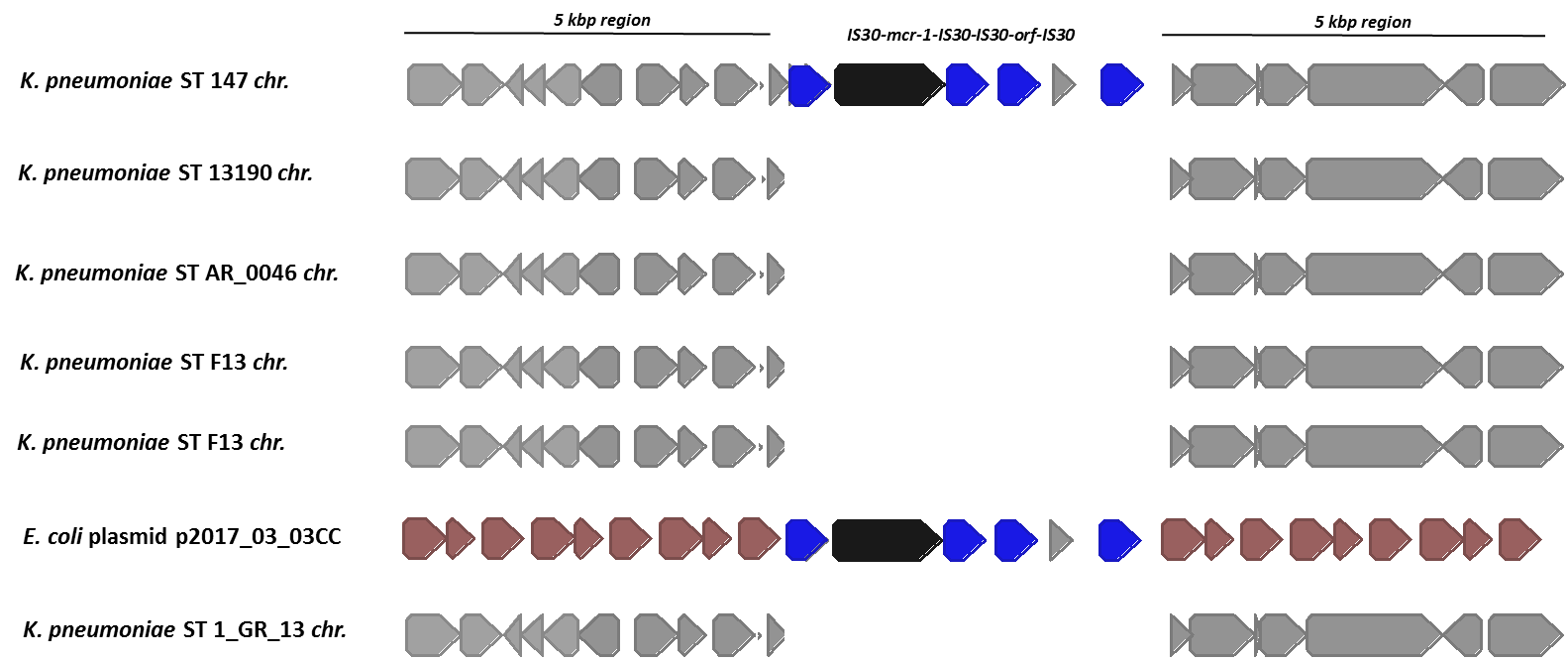

Supplement: Supplementary Figure 1 — Organization of the chromosomal region containing mcr-1 in ST147 K. pneumoniae. [file Data_Sheet_1.zip › Figure 1.DOCX]
