# Supplementary material for: Genetic Analysis of mcr-1-Carrying Plasmids From Gram-Negative Bacteria in a Dutch Tertiary Care Hospital: Evidence for Intrapatient and Interspecies Transmission Events
Source: Front Microbiol. 2021 Sep 6;12:727435. doi: 10.3389/fmicb.2021.727435 (PMC8450869; doi:10.3389/fmicb.2021.727435)
Supplement: Supplementary Figure 1 — Organization of the chromosomal region containing mcr-1 in ST147 K. pneumoniae. [file Data_Sheet_1.zip › File 1.DOCX]

**Supplementary File 1. Inclusion of isolates and patients from January 2010 until September 2018.**

The graphic representation (File S1, Figure 1) indicates the sample collection process, and the tables (File S1, table 1a and 1b) show the yearly inclusion of bacterial species during the study period.

During the study period (January 2010 until September 2018), the Erasmus MC University Medical Center, Rotterdam, the Netherlands, possessed 1,200 beds, with 97 of these beds being located in the adult or pediatric intensive care units (ICU). Screening for the *mcr-1* gene was only applied to stored (-80°C) clinically relevant highly-resistant microorganisms (HRMO) and non-HRMO isolates known not to be intrinsically resistant to colistin. For this retrospective study, stored (-80°C) HRMO and non-HRMO isolates from different collections, with an MIC >2 µg/mL for colistin, were available for testing for the *mcr-1* gene (Figure 1, table 1).


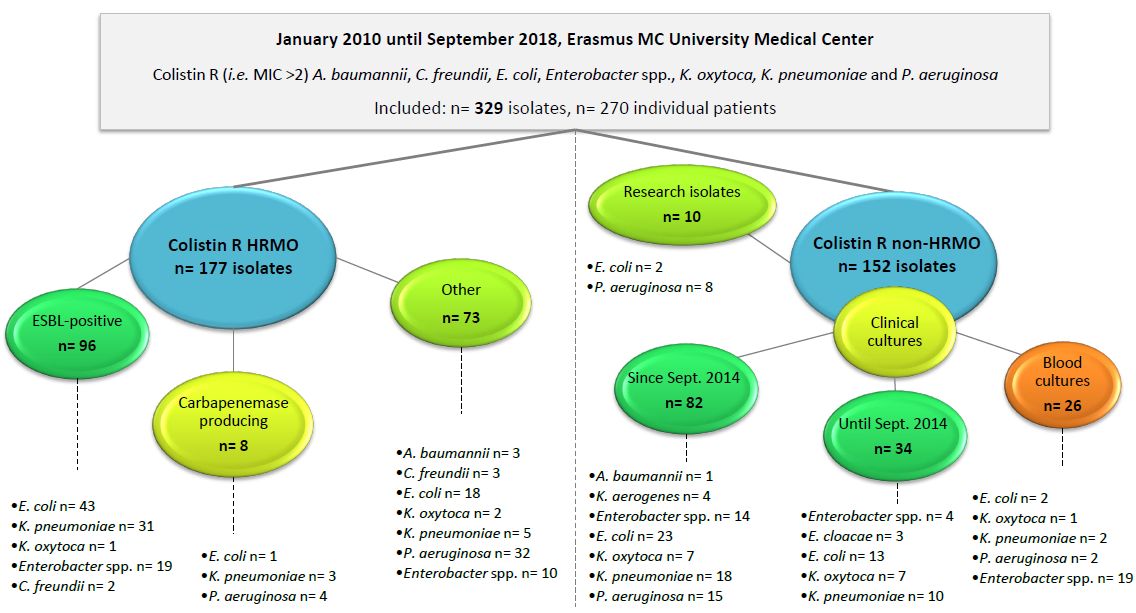


**Figure 1.** Isolate selection and inclusion.

**Abbreviations**: colistin R; colistin resistant, Sept.; September, HRMO; highly-resistant microorganisms, non-HRMO; non highly-resistant microorganisms, ESBL; extended-spectrum beta-lactamase.

First, all HRMO isolates from all possible sample sites (*e.g.* rectum, throat, urine, blood, drain fluid) were stored, and thus available for this study. Second, for non-HRMO only blood isolates were stored (Figure 1). However, since September 2014 non-HRMO colistin resistant isolates tested by VITEK-2 (bioMérieux, Marcy l’Etoile, France) were stored as well (Figure 1). Identification of species was performed with VITEK-2 and Matrix Assisted Laser Desorption Ionization Time of Flight Mass Spectrometry (MALDI-TOF MS; Bruker Daltonik, Bremen, Germany). Extended-spectrum beta-lactamase (ESBL) screening was based on VITEK-2 and confirmation was done with E-test or combination disk diffusion (ROSCO Diagnostica A/S, Taastrup, Denmark). Additionally, for study purposes, colistin-resistant *Escherichia coli* were stored from April 2012 until October 2013, and colistin-resistant *Pseudomonas aeruginosa* were stored since November 2009, and thus available for this study (Figure 1). The available strains were grown on Trypticase Soy Agar II culture plates containing 5% Sheep Blood (BD, Heidelberg, Germany). Cultures were incubated at 35°C for 24 hours.

Accurate antimicrobial susceptibility testing of colistin remains difficult, and the methodology for testing and breakpoints changed over time. Various studies have shown that automated susceptibility testing systems (*e.g.* the VITEK-2 system as used in this study) could give unreliable results. Therefore, we tested an additional 78 *E. coli* and *K. pneumoniae* isolates (71 unique patients) with a colistin MIC of 1 or 2 µg/mL for presence of the *mcr-1* gene. No additional *mcr-1* positive isolates were identified in this collection. Therefore, we only included relevant isolates with a colistin MIC of >2 µg/mL in this study.

**Table 1a.** Inclusion of bacterial species (*n*= 329) during the study period, and the year of isolation.

| **Microorganism** | **2010** | **2011** | **2012** | **2013** | **2014** | **2015** | **2016** | **2017** | **2018** | **Total** |
| --- | --- | --- | --- | --- | --- | --- | --- | --- | --- | --- |
| *Escherichia coli* | 9 | 7 | 5 | 7 | 13 | 11 | 31 | 13 | 6 | **102 (31.0%)** |
| *Klebsiella pneumoniae* | 9 | 12 | 7 | 7 | 7 | 6 | 4 | 11 | 6 | **69 (21.0%)** |
| *Enterobacter* spp. | 9 | 6 | 7 | 6 | 5 | 7 | 6 | 9 | 8 | **63 (19.1%)** |
| *Pseudomonas aeruginosa* | 9 | 7 | 4 | 4 | 5 | 5 | 7 | 12 | 8 | **61 (18.5%)** |
| *Klebsiella oxytoca* | 2 | 4 | 1 | 2 | 2 | 3 | 0 | 2 | 2 | **18 (5.5%)** |
| *Klebsiella aerogenes* | 2 | 0 | 0 | 0 | 1 | 0 | 1 | 1 | 1 | **6 (1.8%)** |
| *Citrobacter freundii* | 0 | 0 | 1 | 0 | 1 | 1 | 0 | 2 | 0 | **5 (1.5%)** |
| *Acinetobacter baumannii* | 0 | 0 | 0 | 0 | 0 | 0 | 3 | 1 | 0 | **4 (1.2%)** |
| *Kluyvera georgiana* | 0 | 0 | 0 | 0 | 0 | 1 | 0 | 0 | 0 | **1 (0.3%)** |
|  | **40 (12.2%)** | **36 (10.9%)** | **25 (7.6%)** | **26 (7.9%)** | **34 (10.3%)** | **34 (10.3%)** | **52 (15.8%)** | **51 (15.5%)** | **31 (9.4%)** | **329 (100%)** |

**Table 1b.** Inclusion of *mcr*-1 positive bacterial species (*n*= 22) during the study period, and the year of isolation.

| **Microorganism** | **2010** | **2011** | **2012** | **2013** | **2014** | **2015** | **2016** | **2017** | **2018** | **Total** |
| --- | --- | --- | --- | --- | --- | --- | --- | --- | --- | --- |
| *Escherichia coli* | 2 | 0 | 0 | 0 | 2 | 4 | 7 | 4 | 0 | **19 (86.4%)** |
| *Klebsiella pneumoniae* | 0 | 0 | 0 | 0 | 0 | 1 | 1 | 0 | 0 | **2 (9.1%)** |
| *Enterobacter* spp. | 0 | 0 | 0 | 0 | 0 | 0 | 0 | 0 | 0 | **0 (0%)** |
| *Pseudomonas aeruginosa* | 0 | 0 | 0 | 0 | 0 | 0 | 0 | 0 | 0 | **0 (0%)** |
| *Klebsiella oxytoca* | 0 | 0 | 0 | 0 | 0 | 0 | 0 | 0 | 0 | **0 (0%)** |
| *Klebsiella aerogenes* | 0 | 0 | 0 | 0 | 0 | 0 | 0 | 0 | 0 | **0 (0%)** |
| *Citrobacter freundii* | 0 | 0 | 0 | 0 | 0 | 0 | 0 | 0 | 0 | **0 (0%)** |
| *Acinetobacter baumannii* | 0 | 0 | 0 | 0 | 0 | 0 | 0 | 0 | 0 | **0 (0%)** |
| *Kluyvera georgiana* | 0 | 0 | 0 | 0 | 0 | 1 | 0 | 0 | 0 | **1 (4.5%)** |
|  | **2 (9.1%)** | **0 (0%)** | **0 (0%)** | **0 (0%)** | **2 (9.1%)** | **6 (27.3%)** | **8 (36.4%)** | **4 (18.2%)** | **0 (0%)** | **22 (100%)** |
