# Supplementary material for: Genetic Analysis of mcr-1-Carrying Plasmids From Gram-Negative Bacteria in a Dutch Tertiary Care Hospital: Evidence for Intrapatient and Interspecies Transmission Events
Source: Front Microbiol. 2021 Sep 6;12:727435. doi: 10.3389/fmicb.2021.727435 (PMC8450869; doi:10.3389/fmicb.2021.727435)
Supplement: Supplementary Figure 1 — Organization of the chromosomal region containing mcr-1 in ST147 K. pneumoniae. [file Data_Sheet_1.zip › Table 1.DOCX]

| **Patient** | | **Year** | **Species** | **Plasmid**  **Incompatibility types** | **Plasmid**  **sizes (bp)** |
| --- | --- | --- | --- | --- | --- |
| A | 2010 | | *E. coli* | IncI1, IncI2, p0111 | 111,682, 58,321, 54,793 |
| B | 2010 | | *E. coli* | IncFIA, IncFIB, IncFII, IncI1, | 4,656, 3,436, 46,283, 59,763 |
| C | 2014 | | *E. coli*♦^2^ | Col, IncFIA, IncFII, IncI1 | 1,386, 54,160, 66,652, 107,472 |
| C | 2014 | | *E. coli*♦^2^ | Col●, IncFIA, IncFII●, IncI1 | 2,101, 47,122, 64,601, 107,472 |
| D | 2015 | | *E. coli*♦^1^ | IncI, IncX1●, p0111● | 116,424, 37,196, 93,855 |
| E | 2015 | | *K. pneumoniae* | IncFIB, IncN2, IncP1● | 10,671, 34,145, 53,908 |
| E | 2015 | | *K. georgiana* | Col●, IncFII●, IncN2● | 2,317, 67,361, 49,588 |
| E | 2015 | | *E. coli* | IncFIB, IncFIC, IncFII, IncHI2, IncHI2A, IncI1, IncN, IncX1 | 28,505, 35,102, 20,064, 27,804, 111,483 90,297, 31,167, 6,578 |
| F | 2015 | | *E. coli* | IncFIB, IncFII, IncQ1, IncY | 70,965, 43,767, 45,597, 51,397 |
| G | 2015 | | *E. coli*♦^3^ | IncFIB, IncFII, IncY | 70,965, 43,767, 51,394 |
| H | 2016 | | *E. coli* | IncFIB | 162,529 |
| H | 2016 | | *E. coli* | IncFIB, IncFIC | 93,412, 61,436 |
| D | 2016 | | *E. coli*♦^1^ | IncFIB, IncI1, IncX1, p0111 | 23,219, 19,520, 36,428, 86,798 |
| D | 2016 | | *E. coli* | IncFIB, IncX1●, p0111● | 136,386, 37,196, 91,823 |
| E | 2016 | | *E. coli* | IncFII, IncI1, IncP1●, IncX1, IncY | 52,379, 97,004, 53,908, 4,890, 41,343 |
| I | 2016 | | *E. coli* | IncFII | 163,517 |
| G | 2016 | | *E. coli*♦^3^ | Col, IncFIB, IncFII, IncHI2A, IncX4● | 3,357, 38,945, 36,443, 12,243, 28,688 |
| N | 2016 | | *K. pneumoniae* | Col●, IncX1● | 7,334, 38,623 |
| J | 2017 | | *E. coli* | IncB, IncFIB, IncFII, IncN● | 92,647, 8,539, 18,523, 41,402 |
| K | 2017 | | *E. coli* | Col●, IncFIA, IncFII, IncX1 | 1,551, 55,003, 72,687, 33,662 |
| L | 2017 | | *E. coli* | IncFIB●, IncI1●, p0111● | 118,598, 96,453, 98,784 |
| M | 2017 | | *E. coli* | Col●, IncFIA/IncFIB, IncFIB, IncFII●, IncI1, IncI2 | 1,454, 90,096, 8,406, 85,476, 87,751, 51,634 |

**Supplementary Table 1: Overview of non *mcr-1* plasmids including size and Inc types.** Genotypically indistinguishable isolates are defined by corresponding superscript numbers next to the species name, complete circular plasmid sequences are indicated with an ● symbol. Plasmid size are shown in the same order as the Inc types.
