# Supplementary material for: Genetic Analysis of mcr-1-Carrying Plasmids From Gram-Negative Bacteria in a Dutch Tertiary Care Hospital: Evidence for Intrapatient and Interspecies Transmission Events
Source: Front Microbiol. 2021 Sep 6;12:727435. doi: 10.3389/fmicb.2021.727435 (PMC8450869; doi:10.3389/fmicb.2021.727435)
Supplement: Supplementary Figure 1 — Organization of the chromosomal region containing mcr-1 in ST147 K. pneumoniae. [file Data_Sheet_1.zip › Table 2.DOCX]

| **Plasmid nr.** | **Genes** | **Antibiotic class** |
| --- | --- | --- |
| 13 | CTX-M beta-lactamase | cephalosporin |
| 14 | CTX-M beta-lactamase | cephalosporin |
| 6 | CTX-M beta-lactamase | cephalosporin |
| 17 | CTX-M beta-lactamase | cephalosporin |
| 19 | CTX-M beta-lactamase | cephalosporin |
|  | fosfomycin thiol transferase | fosfomycin |
| 2 | small multidrug resistance (SMR) antibiotic efflux pump | fluoroquinolone antibiotic |
|  | chloramphenicol acetyltransferase (CAT) | phenicol antibiotic |
|  | APH(3') | aminoglycoside antibiotic |
|  | aadA | aminoglycoside antibiotic |
|  | trimethoprim resistant dihydrofolate reductase dfr | diaminopyrimidine antibiotic |
|  | sulfonamide resistant sul | sulfonamide antibiotic; sulfone antibiotic |
|  | sulfonamide resistant sul | sulfonamide antibiotic; sulfone antibiotic |
|  | aadA | aminoglycoside antibiotic |
|  | APH(6) | aminoglycoside antibiotic |
|  | APH(3'') | aminoglycoside antibiotic |
|  | chloramphenicol acetyltransferase (CAT) | phenicol antibiotic |
|  | MCR phosphoethanolamine transferase | peptide antibiotic |
|  | AAC(3) | aminoglycoside antibiotic |
|  | major facilitator superfamily (MFS) antibiotic efflux pump | tetracycline antibiotic |
|  | APH(6) | aminoglycoside antibiotic |
|  | sulfonamide resistant sul | sulfonamide antibiotic; sulfone antibiotic |
|  | major facilitator superfamily (MFS) antibiotic efflux pump | phenicol antibiotic |
|  | TEM beta-lactamase | monobactam; penem; penam; cephalosporin |
|  | APH(3'') | aminoglycoside antibiotic |
|  | major facilitator superfamily (MFS) antibiotic efflux pump | macrolide antibiotic |
|  | aadA | aminoglycoside antibiotic |
| 20 | AMR Gene Family | aminoglycoside antibiotic |
|  | aadA5 | monobactam; penem; penam; cephalosporin |
|  | TEM beta-lactamase | macrolide antibiotic |
|  | macrolide phosphotransferase (MPH) | sulfonamide antibiotic; sulfone antibiotic |
|  | sulfonamide resistant sul | diaminopyrimidine antibiotic |
|  | trimethoprim resistant dihydrofolate reductase dfr | peptide antibiotic |
|  | MCR phosphoethanolamine transferase | tetracycline antibiotic |
|  | major facilitator superfamily (MFS) antibiotic efflux pump | phenicol antibiotic |

**Supplementary Table 2: Table showing *mcr-1* plasmids that contained additional antibiotic resistance genes and the corresponding antibiotic classes.**
