# Supplementary material for: Genetic Analysis of mcr-1-Carrying Plasmids From Gram-Negative Bacteria in a Dutch Tertiary Care Hospital: Evidence for Intrapatient and Interspecies Transmission Events
Source: Front Microbiol. 2021 Sep 6;12:727435. doi: 10.3389/fmicb.2021.727435 (PMC8450869; doi:10.3389/fmicb.2021.727435)
Supplement: Supplementary Figure 1 — Organization of the chromosomal region containing mcr-1 in ST147 K. pneumoniae. [file Data_Sheet_1.zip › Table 3.DOCX]

**Supplementary Table 3: Additional AMR genes present in ST147 *K. pneumoniae* carrying *mcr-1* in its chromosome.**

| **AMR gene** | **Antibiotic class** |
| --- | --- |
| PmrF | peptide antibiotic |
| CRP | macrolide antibiotic; fluoroquinolone antibiotic; penam |
| Klebsiella pneumoniae KpnH | macrolide antibiotic; fluoroquinolone antibiotic; aminoglycoside antibiotic; carbapenem; cephalosporin; penam; peptide antibiotic; penem |
| Klebsiella pneumoniae KpnG | macrolide antibiotic; fluoroquinolone antibiotic; aminoglycoside antibiotic; carbapenem; cephalosporin; penam; peptide antibiotic; penem |
| emrR | fluoroquinolone antibiotic |
| oqxB | fluoroquinolone antibiotic; glycylcycline; tetracycline antibiotic; diaminopyrimidine antibiotic; nitrofuran antibiotic |
| oqxA | fluoroquinolone antibiotic; glycylcycline; tetracycline antibiotic; diaminopyrimidine antibiotic; nitrofuran antibiotic |
| baeR | aminoglycoside antibiotic; aminocoumarin antibiotic |
| mdtC | aminocoumarin antibiotic |
| mdtB | aminocoumarin antibiotic |
| marA | fluoroquinolone antibiotic; monobactam; carbapenem; cephalosporin; glycylcycline; cephamycin; penam; tetracycline antibiotic; rifamycin antibiotic; phenicol antibiotic; triclosan; penem |
| SHV-11 | carbapenem; cephalosporin; penam |
| Klebsiella pneumoniae KpnF | macrolide antibiotic; aminoglycoside antibiotic; cephalosporin; tetracycline antibiotic; peptide antibiotic; rifamycin antibiotic |
| Klebsiella pneumoniae KpnE | macrolide antibiotic; aminoglycoside antibiotic; cephalosporin; tetracycline antibiotic; peptide antibiotic; rifamycin antibiotic |
| Klebsiella pneumoniae OmpK37 | monobactam; carbapenem; cephalosporin; cephamycin; penam; penem |
| msbA | nitroimidazole antibiotic |
| Escherichia coli mdfA | tetracycline antibiotic; benzalkonium chloride; rhodamine |
| Klebsiella pneumoniae acrA | fluoroquinolone antibiotic; cephalosporin; glycylcycline; penam; tetracycline antibiotic; rifamycin antibiotic; phenicol antibiotic; triclosan |
| acrB | fluoroquinolone antibiotic; cephalosporin; glycylcycline; penam; tetracycline antibiotic; rifamycin antibiotic; phenicol antibiotic; triclosan |
| Escherichia coli ampH beta-lactamase | cephalosporin; penam |
| FosA6 | fosfomycin |
| MCR-1.1 | peptide antibiotic |
| catI | phenicol antibiotic |
| APH(3')-Ia | aminoglycoside antibiotic |
| Brucella suis mprF | peptide antibiotic |
| CBP-1 | penam |
| Escherichia coli UhpT with mutation conferring resistance to fosfomycin | fosfomycin |
| Escherichia coli EF-Tu mutants conferring resistance to Pulvomycin | elfamycin antibiotic |
| Escherichia coli parC conferring resistance to fluoroquinolone | fluoroquinolone antibiotic |
| Escherichia coli gyrA conferring resistance to fluoroquinolones | fluoroquinolone antibiotic |
| Haemophilus influenzae PBP3 conferring resistance to beta-lactam antibiotics | cephalosporin; cephamycin; penam |
| Escherichia coli EF-Tu mutants conferring resistance to Pulvomycin | elfamycin antibiotic |
| Escherichia coli marR mutant conferring antibiotic resistance | fluoroquinolone antibiotic; cephalosporin; glycylcycline; penam; tetracycline antibiotic; rifamycin antibiotic; phenicol antibiotic; triclosan |
