# Supplementary material for: Genetic Analysis of mcr-1-Carrying Plasmids From Gram-Negative Bacteria in a Dutch Tertiary Care Hospital: Evidence for Intrapatient and Interspecies Transmission Events
Source: Front Microbiol. 2021 Sep 6;12:727435. doi: 10.3389/fmicb.2021.727435 (PMC8450869; doi:10.3389/fmicb.2021.727435)
Supplement: Supplementary Figure 1 — Organization of the chromosomal region containing mcr-1 in ST147 K. pneumoniae. [file Data_Sheet_1.zip › Table 4.DOCX]

**Supplementary Table 4. Overview of insertion sequences detected in the plasmids of this study as detected by ISfinder.** Numbers indicate the count of that IS element in the plasmid sequence. Of these, boldface numbers in red indicate the number flanking the *mcr-1* gene (distance ≤ 3 kbp).

| **Inc types >** | IncI2(delta) | | | | | | | | | IncX4 | | | | | | InHI2 or double Inc type | | | | | |
| --- | --- | --- | --- | --- | --- | --- | --- | --- | --- | --- | --- | --- | --- | --- | --- | --- | --- | --- | --- | --- | --- |
| **Plasmid Number >** | 5 | 6 | 7 | 8 | 11 | 12 | 13 | 14 | 15 | 1 | 3 | 4 | 16 | 18 | 21 | 2 | 9 | 10 | 17 | 19 | 20 |
| ISApl1_IS30 |  |  |  |  |  |  |  |  |  |  |  |  |  |  |  | 5(**2**) | **1** | 2(**1**) |  | 3**(1)** | **1** |
| IS26_IS6 |  |  |  |  |  |  |  |  |  | 1 | 1 | 1 | 1 | 1 | 1 |  |  | 4 |  |  | 2 |
| IS3_IS3_IS3 |  |  |  |  |  |  |  |  |  |  |  |  |  |  |  | 1 |  |  |  |  | 1 |
| IS1A_IS1 |  |  |  |  |  |  |  |  |  |  |  |  |  |  |  | 2 |  | 2 |  |  | 2 |
| ISStma11_ISL3 |  |  |  |  |  |  |  |  |  |  |  |  |  |  |  | 1 |  |  |  |  |  |
| IS1133_IS3_IS3 |  |  |  |  |  |  |  |  |  |  |  |  |  |  |  | 1 |  | 1 |  |  |  |
| IS15_IS6 |  |  |  |  |  |  |  |  |  |  |  |  |  |  |  | 2 |  |  |  |  | 3 |
| IS102_IS5_IS903 |  |  |  |  |  |  |  |  |  |  |  |  |  |  |  | 1 |  | 2 |  |  |  |
| ISAba14_IS3_IS150 |  |  |  |  |  |  |  |  |  |  |  |  |  |  |  | 1 |  |  |  |  |  |
| ISKpn12_IS5_IS427 |  |  |  |  |  |  |  |  |  |  |  |  |  |  |  | 1 |  | 1 |  |  |  |
| ISAzo18_IS3_IS150 |  |  |  |  |  |  |  |  |  |  |  |  |  |  |  | 1 |  |  |  |  |  |
| ISEnca1_IS1380 | 1 |  | 1 | 1 |  |  | 1 | 1 |  |  |  |  |  |  |  |  |  |  |  |  |  |
| IS1230B_IS3_IS3 |  |  |  |  |  |  |  |  |  |  |  |  |  |  |  |  |  | 1 |  |  |  |
| IS5D_IS5_IS5 |  |  |  |  |  |  |  |  |  |  |  |  |  |  |  |  |  | 1 |  |  |  |
| ISEc68_IS5_IS5 |  |  |  |  |  |  |  |  |  |  |  |  |  |  |  |  |  | 1 |  |  |  |
| ISKpn26_IS5_IS5 |  |  |  |  |  |  |  |  |  |  |  |  |  |  |  |  |  | 1 |  |  |  |
| IS1326_IS21 |  |  |  |  |  |  |  |  |  |  |  |  |  |  |  |  |  | 1 |  |  |  |
| ISKpn11_IS3_IS150 |  |  |  |  |  |  |  |  |  |  |  |  |  |  |  |  |  | 1 |  |  |  |
| IS15DIV_IS6 |  |  |  |  |  |  |  |  |  |  |  |  |  |  |  |  |  | 1 |  |  | 3 |
| ISEc36_IS3_IS2 |  |  |  |  |  |  |  | 1 |  |  |  |  |  |  |  |  |  |  |  | 1 |  |
| IS2_IS3_IS2 |  |  |  |  |  |  |  |  |  |  |  |  |  |  |  |  |  |  |  | 1 |  |
| ISKpn8_IS3_IS150 |  |  |  |  |  |  |  |  |  |  |  |  |  |  |  |  |  |  |  | 1 |  |
| IS103_IS3_IS150 |  |  |  |  |  |  |  |  |  |  |  |  |  |  |  |  |  |  |  | 1 | 1 |
| IS421_IS4_IS231 |  |  |  |  |  |  |  |  |  |  |  |  |  |  |  |  |  |  |  | 1 |  |
| IS186A_IS4_IS231 |  |  |  |  |  |  |  |  |  |  |  |  |  |  |  |  |  |  |  | 1 | 3 |
| IS1X3_IS1 |  |  |  |  |  |  |  |  |  |  |  |  |  |  |  |  |  |  |  |  | 1 |
| IS6100_IS6 |  |  |  |  |  |  |  |  |  |  |  |  |  |  |  |  |  |  |  |  | 1 |
| IS21_IS21 |  |  |  |  |  |  |  |  |  |  |  |  |  |  |  |  |  |  |  |  | 1 |
| ISEc62_IS21 |  |  |  |  |  |  |  |  |  |  |  |  |  |  |  |  |  |  |  |  | 1 |
| IS1203E_IS3_IS51 |  |  |  |  |  |  |  |  |  |  |  |  |  |  |  |  |  |  |  |  | 1 |
| **Total** | 1 | 0 | 1 | 1 | 0 | 0 | 1 | 2 | 0 | 1 | 1 | 1 | 1 | 1 | 1 | 16 | 1 | 19 | 0 | 9 | 21 |
